# Investigation of *Toxoplasma* infection in zoo animals using multispecies ELISA and GRA7 nested PCR

**DOI:** 10.1186/s12917-022-03425-y

**Published:** 2022-09-06

**Authors:** Ru-Min Liu, Wei-Hsiang Huang, Shang-Lin Wang, Sin-Ling Wang, Pei-Yun Huang, Chen-Yeh Lien, Yen-Hsueh Lai, Pao-Jung Wang, Li-Hsin Wu, Albert Taiching Liao

**Affiliations:** 1 Department of Veterinary Medicine, National Taiwan University, 1 Sec 4 Roosevelt Rd, Taipei, 106216 Taiwan, Republic of China; 2 Graduate Institute of Molecular and Comparative Pathobiology, National Taiwan University, 1 Sec 4 Roosevelt Rd, Taipei, 106216 Taiwan, Republic of China; 3 Institute of Veterinary Clinical Science, National Taiwan University, 1 Sec 4 Roosevelt Rd, Taipei, 106216 Taiwan, Republic of China; 4 Conservation and Research Center, Taipei Zoo, 30 Sec 2 Xinguang Rd, Taipei, 116016 Taiwan, Republic of China; 5 Veterinary Office, Taipei Zoo, 30 Sec 2 Xinguang Rd, Taipei, 116016 Taiwan, Republic of China

**Keywords:** *Toxoplasma*, Zoo animals, ELISA, LAT, Nested PCR, GRA7, B1

## Abstract

**Background:**

*Toxoplasma* is an obligate intracellular protozoan that causes an important zoonotic disease with a worldwide distribution. Felids are the definitive hosts of this parasite, while virtually all warm-blooded animals, including birds, serve as intermediate hosts. Four ring-tailed lemurs (*Lemur catta*) in the Taipei Zoo died of acute *Toxoplasma* infection in June 2019. Since then, *Toxoplasma* has occasionally been identified in this Zoo during necropsy of dead animals and PCR of animal blood samples. Therefore, a general survey of *Toxoplasma* infection in animals in the Zoo seems to be needed.

**Methods and results:**

An indirect multispecies ELISA was used for the first time to screen for *Toxoplasma* infection in 326 serum samples collected from 75 species of animals. The infection rate of *Toxoplasma* was 27% (88/326). A commercial latex agglutination (LAT) assay was used to re-examine the samples with doubtful and uncertain ELISA results (151 samples from 42 species). The infection rate increased to 36.2% (118/326), and the indirect multispecies ELISA appeared to be applicable to 31 of 75 species animals included in this study. Nested PCR assays targeting the dense granule protein 7 (GRA7) gene and B1 gene were also used to detect *Toxoplasma* in DNA samples extracted from 10 liver or blood specimens from 8 animals. GRA7 gene fragments were amplified from 8 samples from 7 animals, while B1 gene fragments were amplified from only 4 samples from 4 animals. From the B1 nested PCR and the sequence data of GRA7 fragments amplified from infectious specimens, the animals in the Zoo were speculated to have been infected by at least three different *Toxoplasma* variants.

**Conclusions:**

According to the serological investigation, we speculated that over one-third (36.2%) of animals in Taipei Zoo presented the infection of *Toxoplasma*, and the indirect multispecies ELISA we used can be applied to detect *Toxoplasma* infection in 31 animal species included in this study. Sequence analysis revealed that at least three *Toxoplasma* variants were infecting the animals of Taipei Zoo.

**Supplementary Information:**

The online version contains supplementary material available at 10.1186/s12917-022-03425-y.

## Background

*Toxoplasma* is an obligate intracellular protozoan that causes an important disease, toxoplasmosis [1]. Virtually all warm-blooded animals, including humans and birds, can be infected by *Toxoplasma*, making it one of the important parasites affecting public health and animal husbandry worldwide [2]. Even so, most warm-blooded animals serve merely as intermediate hosts, and felids are the sole definitive hosts of this parasite [3].

Infection of *Toxoplasma* is contracted mainly by ingesting undercooked or raw meat containing viable tissue cysts or by ingesting food or water contaminated with oocysts. After ingestion, *Toxoplasma* first multiplies asexually in the intestinal epithelia and then spreads through blood circulation to peripheral tissues, including the brain, liver, muscle, and spleen. Infection can also be transmitted through the placenta or via transplantation or transfusion [2]. In the intestinal epithelia of felids, *Toxoplasma* also multiplies sexually to produce oocysts, which are shed in the excrement to contaminate the environment [4].

*Toxoplasma* infection can cause abortion and stillbirth in pregnant animals, especially in swine, sheep, and goats [5–7]. This causes considerable economic losses and is a serious public health concern [8]. In particular, owners of cats as companion animals may acquire *Toxoplasma* infections from their pets. This is a serious threat for women in the first trimester of pregnancy [4]. In the wild, infected animals transmit *Toxoplasma* to the predators that eat them. Moreover, infected felids in the wild may be an important vector of *Toxoplasma* since their excrement contaminates the areas they walk in. Among Zoo animals, most have no symptoms when they become infected. However, *Toxoplasma* causes high levels of acute mortality in several animals, including lemurs (especially ring-tailed lemurs), New World primates, Australiasian marsupials, Herpestidae, some sea mammals, and Passeriformes [9–11].

The Taipei Zoo is the largest Zoo in Taiwan and a popular educational and recreational attraction in northern Taiwan. In June 2019, four captive lemurs (*Lemur catta*) died suddenly within the span of several days. Later, *Toxoplasma* infection was confirmed by histopathological examination following necropsy (paper in preparation). Since then, *Toxoplasma* has occasionally been found in various tissues of dead animals during histopathological examination. *Toxoplasma* DNA has also occasionally been identified in the blood drawn from the animals during health checks. Thus, a large-scale screen to identify *Toxoplasma* infections in captive animals seems to be urgent and important for this Zoo.

To carry out a large-scale investigate *Toxoplasma* infection in the Zoo, a multispecies *Toxoplasma* ELISA kit was introduced to screen for *Toxoplasma* infection, and a commercial LAT (latex agglutination test) assay was used to re-examine the samples with uncertain results in ELISA test. Nested PCR (nPCR) assays targeting the GRA7 gene and B1 gene were also used to detect and genotype *Toxoplasma* in the tissues and blood collected from several sick and dead animals.

## Results

### Elisa

The ELISA results for 326 serum samples are listed in Table 1. After ELISA examination, 27% (88/326) of serum samples were interpreted as positive (P), while 71.8% (234/326) of serum samples were deemed negative (N). The remaining serum samples (1.2%; 4/326) were determined to be doubtful (D). The doubtful serum samples were reexamined using LAT to determine the result of *Toxoplasma* infection in those serum samples. In addition, the ELISA kit used in this study has not previously been applied to detect *Toxoplasma* infection in most of the species included in this study. It (ELISA kit) may not applicable for some of the species included in this study and causes false positive/ negative results in all the samples collected from those species. If the ELISA results of samples collected from the same species were all positive (ex. *C. dromedaries* and *T. indicus* in Table 1) or all negative (ex. *P. salvania* and *A. fulgens* in Table 1), they were considered uncertain results and were also re-examined by LAT. Thus, the ELISA results of 27 samples from 14 species (all positive) and 120 samples from 34 species (all negative) were deemed uncertain results, respectively.

**Table 1:** The results of ELISA examination and the adjusted results after LAT examination

Order/ Family	Genus	Species	P	N	D	AP	AN
*Artiodactyla* (96)							
*Bovidae* (48)	*Addax* (11)	*A. nasomaculatus* (11)	1	10	**–**	1	10
	*Ammotragus* (2)	*A. lervia* (2)	1	1	**–**	1	1
	*Naemorhedus* (8)	*N. swinhoei* (8)	6	2	**–**	6	2
	*Ovis* (8)	*O.canadensis* (8)	2	5	1	3	5
	*Oryx* (2)	*O. beisa* (1)	1	–	–	1	–
		*O. gazelle* (1)	–	1		–	1
	*Taurotragus* (8)	*T. oryx* (8)	1	7	**–**	1	7
	*Tragelaphus* (9)	*T. angasii* (1)	–	1	–	–	1
		*T. eurycerus* (8)	1	7	–	1	7
*Camelidae* (7)	*Camelus* (5)	*C. bactrianus* (2)	1	1		1	1
		*C. dromedaries* (3)	3	–	–	3	–
	*Vicugna* (2)	*V. pacos* (2)	2	–	**–**	2	–
*Cervidae* (23)	*Cervus* (3)	*C. nippon* (3)	1	2	**–**	1	2
	*Muntiacus* (20)	*M. reevesi* (20)	12	7	1	12	8
*Giraffidae* (1)	*Giraffa* (1)	*G. camelopardalis* (1)	1	–	**–**	1	–
*Suidae* (17)	*Porcula* (5)	*P. salvania* (5)	–	5	**–**	2	3
	*Sus* (12)	*S. scrofa* (12)	5	7	**–**	5	7
*Carnivora* (109)							
*Ailuridae* (10)	*Ailurus* (10)	*A. fulgens* (10)	–	10	**–**	–	10
*Canidae* (2)	*Canis* (2)	*C. lupus* (2)	2	–	**–**	2	–
*Felidae* (23)	*Lynx* (2)	*L. lynx* (1)	1	–	–	1	–
		*L. rufus* (1)	–	1	–	1	–
	*Neofelis* (1)	*N. nebulosi* (1)	–	1	**–**	1	–
	*Panthera* (8)	*P. leo* (3)	2	1	–	2	1
		*P. pardus* (1)	–	–	1	1	–
		*P. tigris tigris* (4)	1	3	–	1	3
	*Prionailurus* (8)	*P. bengalensis* (8)	4	4	**–**	4	4
	*Puma* (4)	*P. concolor* (4)	2	2	**–**	2	2
*Herpestidae* (8)	*Suricata* (8)	*S. suricatta* (8)	–	8	**–**	5	3
*Hyaenidae* (2)	*Crocuta* (2)	*C. crocuta* (2)	–	2	**–**	2	–
*Mustelidae* (16)	*Aonyx* (14)	*A. cinereal* (14)	–	14	**–**	1	13
	*Melogale* (2)	*M. moschata* (2)	–	2	**–**	–	2
*Procyonidae* (10)	*Nasuella* (7)	*N. olivacea* (7)	1	6	**–**	1	6
	*Potos* (3)	*P. flavus* (3)	–	3	**–**	–	3
*Ursidae* (23)	*Ailuropoda* (2)	*A. melanoleuca* (2)	–	2	**–**	1	1
	*Helarctos* (4)	*H. malayanus* (4)	2	2	**–**	2	2
	*Ursus* (17)	*U. arctos* (2)	2	–	–	2	–
		*U. thibetanus* (15)	4	11	–	4	11
*Viverridae* (15)	*Paguma* (13)	*P. larvata* (13)	–	13	**–**	4	9
	*Viverricula* (2)	*V. indica* (2)	–	2	**–**	1	1
*Diprotodontia* (10)							
*Phascolarctidae* (8)	*Phascolarctos* (8)	*P. cinereus* (8)	–	8	**–**	4	4
*Macropodidae* (2)	*Macropus* (1)	*M. giganteus* (1)	–	–	1	1	–
	*Thylogale* (1)	*T. billardierii* (1)	1	–	**–**	1	–
*Perissodactyla* (31)							
*Tapiridae* (6)	*Tapirus* (6)	*T. indicus* (6)	6	–	**–**	6	–
*Equidae* (19)	*Equus* (19)	*E. africanus* (9)	1	8	–	1	8
		*E. ferus* (4)	1	3	–	1	3
		*E. ferus caballus* (4)	–	4	–	4	–
		*E. quagga* (2)	–	2	–	1	1
*Rhinocerotidae* (6)	*Ceratotherium* (6)	*C. simum* (6)	5	1	**–**	5	1
*Pholidota* (1)							
*Manidae* (1)	*Manis* (1)	*M. pentadactyla* (1)	–	1	–	1	–
*Pilosa* (13)							
*Choloepodidae* (9)	*Choloepus* (9)	*C. didactylus* (9)	2	7	–	2	7
*Myrmecophagidae* (4)	*Myrmecophaga* (1)	*M. tridactyla* (1)	–	1	–	1	–
	*Tamandua* (3)	*T. tetradactyla* (3)	–	3	–	–	3
*Primates* (58)							
*Atelidae* (7)	*Ateles* (7)	*A. hybridus* (5)	–	5	–	–	5
		*A. geoffroyi* (2)	2	–	–	2	–
*Cebidae* (4)	*Callithrix* (2)	*C. aurita* (2)	–	2	**–**	–	2
	*Cebus* (1)	*C. capucinus* (1)	1	–	**–**	1	–
	*Saimiri* (1)	*S. sciureus* (1)	–	1	**–**	–	1
*Cercopithecidae* (6)	*Erythrocebus* (1)	*E. patas* (1)	–	1	**–**	–	1
	*Macaca* (3)	*M. cyclopis* (1)	–	1	–	–	1
		*M. nemestrina* (2)	–	2	–	–	2
	*Papio* (2)	*P. anubis* (2)	–	2	**–**	–	2
*Daubentoniidae* (1)	*Daubentonia* (1)	*D. madagascariensis* (1)	–	1	**–**	–	1
*Hominidae* (10)	*Gorilla* (2)	*G. gorilla* (2)	–	2	**–**	–	2
	*Pan* (5)	*P. troglodytes* (5)	2	3	**–**	2	3
	*Pongo* (3)	*P. pygmaeus* (3)	1	2	**–**	1	2
*Hylobatidae* (3)	*Hylobates* (2)	*H. lar* (2)	–	2	**–**	–	2
	*Symphalangus* (1)	*S. syndactylus* (1)	1	–	**–**	1	–
*Lemuridae* (26)	*Eulemur* (3)	*E. fulvus* (3)	1	2	**–**	1	2
	*Lemur* (12)	*L. catta* (12)	–	12	**–**	–	12
	*Varecia* (11)	*V. variegate* (11)	1	10	**–**	1	10
*Lorisidae* (1)	*Nycticebus* (1)	*N. pygmaeus* (1)	–	1	**–**	–	1
*Proboscidea* (4)							
*Elephantidae* (4)	*Elephas* (2)	*E. maximus* (2)	2	–	**–**	–	2
	*Loxodonta* (2)	*L. africana* (2)	2	–	**–**	–	2
*Rodentia* (4)							
*Castoridae* (3)	*Castor* (3)	*C. canadensis* (3)	–	3	**–**	1	2
*Sciuridae* (1)	*Cynomys* (1)	*C. ludovicianus* (1)	–	1	**–**	1	–
Total	*–*	326	88	234	4	118	208
%	*–*	–	27	71.8	1.2	36.2	63.8

*P* Positive; *N* Negative; *D* Doubtful; *AP* Adjusted positive after LAT assay; *AN* Adjusted negative after LAT assay

### LAT (latex agglutination test)

The serum samples with doubtful (4 samples from 4 species) and uncertain (147 samples from 48 species) results in ELISA test were re-examined by LAT. In the samples (27) whose ELISAs were all positive in the same species, the LAT results for most samples matched the ELISA results, except for 4 samples (4/27 = 14.8%) collected from 2 African elephants (*L. africana)* and 2 Asian elephants (*E. maximus*), which were negative (Table 2). However, the results of LAT did not match the ELISA results for 18 of 34 species whose ELISA results were all negative. In addition, one (*Formosan muntjac*) of four doubtful samples in ELISA was determined to be positive, and the remaining samples were negative. The results of LAT were used to adjust the ELISA results for all samples, and the results shown in Table 1 were reassigned as adjusted positive (AP) and adjusted negative (AN). After adjustment, the infection rate of *Toxoplasma* was 36.2% (108/326).

**Table 2:** The results of LAT assay for the samples with uncertain and doubtful results in ELISA assay

Species name (common name)	P	N	Subtotal
**Uncertain – all positive**^**a**^			
*A. geoffroyi* (Black Spider Monkey)	2	–	2
*C. capucinus* (Capuchin Monkey)	1	–	1
*C. dromedarius* (Dromedary)	3	–	3
*C. lupus* (Wolf)	2	–	2
*E. maximus* (Asian Elephant) ^c^	–	2	2
*G. camelopardalis* (Giraffe)	1	–	1
*L. africana* (African Elephant) ^c^	–	2	2
*L. lynx* (Eurasian Lynx)	1	–	1
*O. beisa* (East African right-angled antelope)	1	–	1
*S. syndactylus* (Siamang)	1	–	1
*T. billardierii* (Red-bellied Kangaroo)	1	–	1
*T. indicus* (Malay Tapir)	6	–	6
*U. arctos* (Brown Bear)	2	–	2
*V. pacos* (Alpaca)	2	–	2
**Uncertain - all negative**^**b**^			
*A. cinerea* (Asian small-clawed otter) ^c^	1	13	14
*A. fulgens* (Himalayan Red Panda)	–	10	10
*A. hybridus* (Brown Spider Monkey)	–	5	5
*A. melanoleuca* (Big Panda) ^c^	1	1	2
*C. aurita* (White-eared Tamarin)	–	2	2
*C. canadensis* (American Beaver) ^c^	1	2	3
*C. crocuta* (Spotted Hyena) ^c^	1	1	2
*C. ludovicianus* (Groundhog) ^c^	1	–	1
*D. madagascariensis* (Finger Monkey)	–	1	1
*E. ferus caballus* (Mini Horse) ^c^	4	–	4
*E. patas* (Red Monkey)	–	1	1
*E. quagga* (Plains Zebra) ^c^	1	1	2
*G. gorilla* (Western Gorilla) ^c^	2	–	2
*H. lar* (White-handed Gibbon)	–	2	2
*L. catta* (Ring-tailed lemur)	–	12	12
*L. rufus* (Censored Cat) ^c^	1	–	1
*M. cyclopis* (Taiwan Macaque)	–	1	1
*M. moschata* (Ferret badger)	–	2	2
*M. nemestrina* (Pig-tailed Macaque)	–	2	2
*M. pentadactyla* (Pangolin) ^c^	1	–	1
*M. tridactyla* (Giant Anteater) ^c^	1	–	1
*N. nebulosa* (Clouded Leopard) ^c^	1	–	1
*N. pygmaeus* (Little Loris)	–	1	1
*O. gazella* (South African Sword Antelope) ^c^	1	–	1
*P. anubis* (East African Baboon)	–	2	2
*P. cinereus* (Koala) ^c^	4	4	8
*P. flavus* (Honey Bear)	–	3	3
*P. larvata* (Civet) ^c^	4	9	13
*P. salvania* (Ji Zhu) ^c^	2	3	5
*S. sciureus* (Squirrel Monkey)	–	1	1
*S. suricatta* (Fox Meng) ^c^	5	3	8
*T. angasii* (An’s Nyala)	–	1	1
*T. tetradactyla* (Little Anteater)	–	3	3
*V. indica* (Little Civet) ^c^	1	1	2
**Doubtful**			
*M. giganteus* (Eastern Wallaby)	1	–	1
*M. reevesi* (Formosan muntjac)	–	1	1
*O. canadensis* (Bighorn sheep)	1	–	1
*P. pardus* (Leopard)	1	–	1
Total	57	94	151

*P* Positive; *N* Negative; ^a^ The ELISA results of all samples from the same species were positive.; ^b^ The ELISA results of all samples from the same species were negative.; ^c^ The results of LAT assay were different from the results of ELISA assay

### GRA7 nPCR and B1 nPCR

**Fig. 1:**
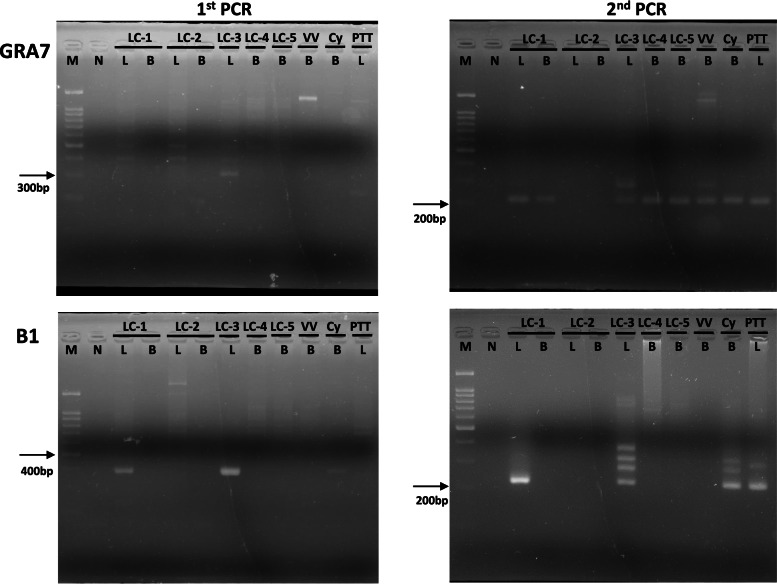
Nested PCR targeted GRA7 and B1 genes of *Toxoplasma*

GRA7 gene fragments could be amplified from most (8/10) of the DNA samples, except for the DNAs extracted from the liver and blood of animal LC-2 (upper right panel, Fig. 1). This reveals that animal LC-2 might be free from *Toxoplasma* infection. Unexpectedly, B1 gene fragments could be amplified only from the DNA samples (4/10) extracted from the livers of animals LC-1, LC-3, and PTT and the blood of animal Cy (lower right panel, Fig. 1). In addition to the DNA samples extracted from the liver and blood of LC-2 animals, we could not amplify any B1 gene fragment from half of the DNA samples (4/8) extracted from the blood of LC-1, LC-4, LC-5, and VV animals. In particular, the GRA7 gene could be detected in the DNA samples extracted from both the liver and blood of animal LC-1, while the B1 gene could be detected in the DNA sample extracted from the liver, but not that from the blood, of animal LC-1. This finding indicates that animals in the Zoo were infected by at least two different *Toxoplasma* variants and that animal LC-1 appeared to be infected by two different *Toxoplasma* variants.

Nested PCR was performed using DNAs extracted from liver or blood of various animals as template. PCR products targeting GRA7 gene and B1 gene were separated on 3% agarose gel. The product sizes of 1st PCR were 316 bp for GRA7 gene (left-upper panel) and 400 bp for B1 gene (left-lower panel). The product sizes of 2nd PCR were 222 bp for GRA7 gene (right-upper panel) and 220 bp for B1 gene (right-lower panel). The GRA7 gene could be detected in the DNA samples of LC-1 (L&B), LC-3(L), LC-4(B), LC-5(B), VV(B), Cy(B) and PTT(L), while the B1 gene could be detected in the DNA samples of LC-1(L), LC-3(L), Cy(B) and PTT(L). M: Marker; N: Negative control; LC-1 ~ LC-5: *Lemur catta*-1 *~ Lemur catta*-5; VV: *Varecia variegate*; Cy: *Cynomys*; PTT: *Panthera tigris tigris*; L: Liver; B: Blood.

### Sequence alignment of GRA7 nPCR products

**Fig. 2:**
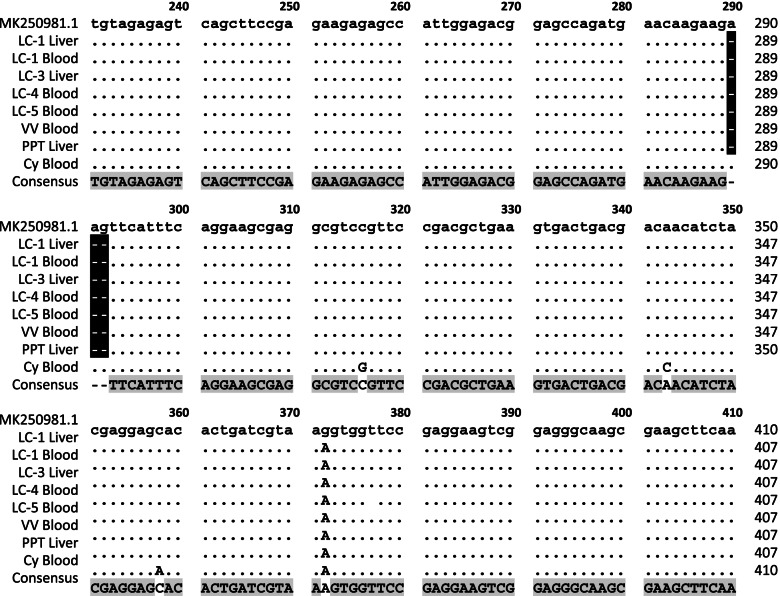
Sequence alignment of GRA7 nPCR products from various samples

To determine the genotype of *Toxoplasma* infecting the animals, the products of GRA7 nPCR were sent for sequencing. The DNA sequences of the GRA7 gene from the RH strain (Accession #: MK250981.1) were used as references. As shown in Fig. 2, animals LC-1 (liver), LC-3 (liver), PTT (liver), and VV (blood) had the same GRA7 sequences. They all lacked three nucleotides (AAG) at nucleotides 290–292 and had one nucleotide replacement (G to A) at nucleotide 372. Animal Cy appeared to be infected with another *Toxoplasma* variant. In addition to the replacement (G to A) at nucleotide 372, three extra replacements were found at nucleotides 316 (C to G), 343 (A to C), and 358 (C to A).

Alignment of GRA7 nPCR products amplified from 10 DNA samples revealed the presence of two different *Toxoplasma* variants in the Zoo animals. The GRA7 sequences (Accession No.: MH250981.1) of RH strain ranged from nucleotide 240 to nucleotide 400 was used as reference sequences. LC-1 ~ LC-5: *Lemur catta*-1 *~ Lemur catta*-5; VV: *Varecia variegate*; Cy: *Cynomys*; PTT: *Panthera tigris tigris*.

## Discussions

ELISA and agglutination assays are both serological tests that are commonly used to detect *Toxoplasma* infection. ELISA requires less time (a couple of hours) and usually has species limitations, while the agglutination assay is a time-consuming method (over 10 hours) but is not species-specific. At the beginning of the study (early 2020), commercialized agglutination kits were not available to us due to the shortage of manufacture and international transport under the COVID-19 pandemic. At that time, the multispecies ELISA kit used in this study was the only assay available to us. Although this ELISA kit has been reported to detect *Toxoplasma* infection in several species [12–14], this study represents the first time it has been used to detect *Toxoplasma* infection in over 70 species of animals in a single study. Another serological assay should be conducted to reconfirm the results of the multispecies ELISA assay in this study, and fortunately, we were able to obtain a commercial LAT kit in early 2021.

Since the results of LAT examination did not match the results of ELISA examination in all cases (Table 2), we categorized the species included in this study into three groups: may recommend (31), need more tests (24), and may not recommend (20) (Table 3). The species (20 species) for which the results of both assays (ELISA and LAT) were different were classified into the “may not recommend” group. The species (24 species) for which test sample number were fewer than three, even if the results of both assays were the same, were classified into the “need more tests” group. The remaining 31 species in this study were classified into the “may recommend” group. Test sample number of those species was more than three, and the results of ELISA assay were not all positive/negative in the same species (22 species), or the results were same in both ELISA and LAT assays (9 species). Still, the sample number of this study for each species was limited. Future studies involving more samples for each species are recommended.

**Table 3:** Applicable animal species of the ID screen® toxoplasmosis indirect multispecies ELISA Kit

The animal species (75 species)			
**May recommend (31 species)**			
*A. fulgens*	*A. hybridus*	*A. lervia*	*A. nasomaculatus*
*C. didactylus*	*C. dromedaries*	*C. nippon*	*C. simum*
*E. africanus*	*E. ferus*	*E. fulvus*	*H. malayanus*
*L. catta*	*M. reevesi*	*N. olivacea*	*N. swinhoei*
*O.canadensis*	*P. bengalensis*	*P. concolor*	*P. flavus*
*P. leo*	*P. pygmaeus*	*P. tigris tigris*	*P. troglodytes*
*S. scrofa*	*T. eurycerus*	*T. indicus*	*T. tetradactyla*
*T. oryx*	*U. thibetanus*	*V. variegata*	
**Need more tests (24 species)**			
*A. geoffroyi*	*C. aurita*	*C. bactrianus*	*C. capucinus*
*C. lupus*	*D. madagascariensis*	*E. patas*	*G. camelopardalis*
*H. lar*	*L. lynx*	*M. cyclopis*	*M. giganteus*
*M. moschata*	*M. nemestrina*	*N. pygmaeus*	*O. beisa*
*P. anubis*	*P. pardus*	*S. sciureus*	*S. syndactylus*
*T. angasii*	*T. billardierii*	*U. arctos*	*V. pacos*
**May not recommend (20 species)**			
*A. cinereal*	*A. melanoleuca*	*C. canadensis*	*C. ludovicianus*
*C. crocuta*	*E. ferus caballus*	*E. quagga*	*E. maximus*
*G. gorilla*	*L. africana*	*L. rufus*	*M. pentadactyla*
*M. tridactyla*	*N. nebulosi*	*O. gazella*	*P. cinereus*
*P. larvata*	*P. salvania*	*S. suricatta*	*V. indica*

The B1 gene, which is present in 35 repeats in the *Toxoplasma* genome*,* was first introduced as a PCR target to detect *Toxoplasma* infection and has been used in many studies since 1989 [15, 16]. In 2000, a 529 bp fragment of the repetitive element (RE) gene was reported. RE is present in 200–300 repeats in the *Toxoplasma* genome, and PCR targeting the RE gene has 10-fold higher sensitivity than PCR targeting the B1 gene [17]. However, later studies mentioned that some genotypes of *Toxoplasma* had lost part or all of the RE gene repeats, and the B1 and RE genes have higher mutation rates among different *Toxoplasma* genotypes compared to another gene, “GRA7”, which is also present in 200–300 repeats in the *Toxoplasma* genome [18]. According to our results in Fig. 1, DNA fragments could be amplified from 8 specimens of 7 animals by GRA7 nPCR but from only 4 specimens of 4 animals by B1 gene nPCR. Some of the *Toxoplasma* variants found in this study seemed to have lost their B1 gene.

*Toxoplasma* infections in a Zoo environment have been reported in two previous studies. Lin and his colleagues used LAT to detect antibodies against *Toxoplasma* from 1107 frozen serum samples collected from 5 species of reptiles, 25 species of birds, and 112 species of mammals [19]. The overall seroprevalence was 38.75%, which is similar to the value in our study (36.2%). Later, another team used B1 semi-nPCR to detect *Toxoplasma* infection in 171 muscle DNA samples that were extracted from the tissues of 89 species of dead animals [20]. There were 14 samples (8.2%) showing suspected positivity, and only two of them could be confirmed by sequencing. The results they obtained were quite different from our findings. According to our results (Fig. 1), choosing the B1 gene as a molecular target might be the reason for their low prevalence of *Toxoplasma* infection.

During the past decades, several studies have investigated *Toxoplasma* infection in animals in Taiwan. These studies included studies in companion animals [21–23], reproductive animals [14, 24], wild birds [25] and Zoo animals [19, 20]. Most of these studies used serological methods to investigate the prevalence of *Toxoplasma* infection, except for one study that used semi-nPCR to detect *Toxoplasma* DNA in frozen specimens from Zoo animals [20]. However, none of them mentioned the genotypes of the detected *Toxoplasma*. To identify the variants of *Toxoplasma* infecting animals in the Zoo, nPCRs targeting the GRA7 and B1 genes were used to detect *Toxoplasma* infection, and amplicons were sent for sequencing. Unfortunately, we did not have enough DNA to perform genotyping of the *Toxoplasma* in this study.

According to the sequence alignment of the GRA7 gene, the *Toxoplasma* infecting the animals in the Zoo was divided into two variants (Fig. 2). Animal Cy was infected by one of the variants. The other variant could be further subdivided into two variants, in which the B1 gene is detectable (liver of animals LC-1, LC3, and PTT) and undetectable (blood of animals LC-1, LC4, LC-5 and VV), respectively (Fig. 1). Therefore, we estimate that there were at least three different *Toxoplasma* variants infecting those Zoo animals. Interestingly, animal LC1, one of four lemurs who died suddenly in June 2019, appeared to be infected by two *Toxoplasma* variants (Fig. 1). On the basis of our results, we speculated that animal LC1 had been living with a latent infection with the variant B1 gene-bearing variant and died after coinfection with the B1 gene-lacking variant.

## Conclusions

According to the serological investigation, we speculated that over one-third (36.2%) of animals in Taipei Zoo presented the infection of *Toxoplasma*, and the indirect multispecies ELISA we used can be applied to detect *Toxoplasma* infection in 31 animal species included in this study. Sequence analysis revealed that at least three *Toxoplasma* variants were infecting the animals of Taipei Zoo.

## Methods

### Samples

All the samples (sera or DNA) used in this study were selected from the frozen specimen archive of the Veterinary Office of the Taipei Zoo. They were collected from dead animals during necropsy or from live animals during routine health checks or examinations of sick animals between January 2019 and May 2021. None of the samples was collected specifically for this study. The ethics committee of Institutional Animal Care and Use Committee of National Taiwan University approved the study (NTU-110-EL-00108). In total, 326 serum samples collected from 9 orders, 33 families, 63 genera, and 75 species were used in this study. They were collected from animals belonging to the following orders (Sample #/family #/genus #/species #): *Pilosa* (13/2/3/3), *Primates* (58/8/17/19), *Artiodactyla* (96/5/14/17), *Perissodactyla* (31/3/3/6), *Carnivora* (109/9/18/22), *Rodentia* (4/2/2/2), *Proboscidea* (4/1/2/2), *Diprotodontia* (10/2/3/3), and *Pholidota* (1/1/1/1). In addition, 10 DNA samples extracted from the liver or blood of 8 animals were offered by the Veterinary Office and used to detect *Toxoplasma* DNA and genotypes in this study. They included 3 liver and 4 blood (7 in total) samples from 5 ring-tailed lemurs (*Lemur catta*, LC), 1 blood sample from a black-and-white ruffed lemur (*Varecia variegata*, VV), 1 blood sample from a prairie dog (*Cynomys,* Cy), and 1 liver sample from a Bengal tiger (*Panthera tigris tigris,* PTT). Specimens of animals LC1, LC2, LC3, Cy and PTT were collected during necropsy. Later, in histopathological examination, *Toxoplasma* infection was identified in animals LC1, LC3 and Cy but not animal LC2 and PTT. Animals LC4, LC5 and VV had clinical signs and survived after treatment.

### ELISA (enzyme-linked immunosorbent assay)

The ID Screen® Toxoplasmosis Indirect Multispecies ELISA kit manufactured by ID. Vet (Grabels, France) was used in this study to detect the IgG titer against *Toxoplasma* (P30 antigen) for various species. The assay was performed in accordance with the manufacturer’s instructions. Briefly, 100 μl of 10x diluted sample sera and controls (positive and negative sera) were added to the well, which was coated with p30 antigen and incubated at room temperature for 45 minutes. The liquid in the well was discarded, and then the well was washed 3 times with 300 μl of Wash Solution each time. One hundred microliters of Conjugate (10-fold diluted with Dilution Buffer 3) was added to the well and incubated at RT for 30 minutes. The liquid in the well was discarded again and the plate was washed 3 times with 300 μl of Wash Solution each time. Then, 100 μl of Substrate Solution was added to the well and incubated in the dark at room temperature for 15 minutes. Finally, 100 μl of Stop Solution was added to the well, and the optical density (O.D.) of each well was read at 450 nm in a Multiskan™ FC Microplate Photometer (Thermo Fisher Scientific, USA) and recorded. To interpret the result, the S/P percentage [S/P%: (sample O.D. - Negative Control O.D.) × 100/(Positive Control O.D. - Negative Control O.D.)] was first calculated for each serum sample. An S/P% greater than or equal to 50% was considered positive, while an S/P less than or equal to 30% was considered negative. If S/P% was between 30 and 50%, the sample was considered doubtful.

### Latex agglutination test (LAT)

The MAST® TOXOREAGENT LAT assay purchased from Mast Group (Liverpool, UK) was used in this study to re-examine the samples with uncertain results in ELISA assay. The examination was conducted in accordance with the manufacturer’s instructions. Briefly, 25 μl of serially diluted (2-fold serial dilution: from 16-fold to 512-fold) serum samples and controls (negative and positive) were added to U-shaped bottom microwell plates. A 25 μl aliquot of latex reagent-conjugated whole worm antigens was added to each well. The contents of each well were mixed by gently tapping all four sides of the plate. The plate was covered with a lid and then incubated at room temperature in the dark for 14–16 hours. The agglutination patterns of each well were read on a horizontal surface at an undisturbed position. If agglutination was found in the dilution fold equal to or over 64x, the serum sample was considered to be positive for *Toxoplasma* infection. If agglutination occurred in a dilution fold equal to or lower than 32x, the serum sample was determined to be negative for *Toxoplasma* infection.

### Nested PCR (nPCR)

Nested PCR targeting the GRA7 gene (GRA7 nPCR) of *Toxoplasma* was used to detect *Toxoplasma* DNA from the tissues of Zoo animals. Nested PCR targeting the B1 gene (B1 nPCR) of *Toxoplasma* was used to confirm the results of GRA7 nPCR. The sequences of primer pairs and expected sizes of all PCR products are listed in Table 4. Two microliters of genomic DNA or the 1st PCR product was used as a template to amplify the GRA7 or B1 gene fragment using Taq DNA Polymerase 2x Master Mix RED (Ampliqon PCR Enzymes & Reagents, Copenhagen, Denmark) with the primer pairs listed in Table 1. PCR (1st and 2nd) was performed for 30 thermal cycles of 94 °C for 30 seconds, 48 °C (1st PCR)/52 °C (2nd PCR) for 30 seconds, and 63 °C for 30 seconds. An initial denaturation at 94 °C for 10 minutes was performed before the thermal cycles, and a final extension at 63 °C for 5 minutes was added at the end of the thermal cycles. All the PCR products were separated on 3% agarose and imaged by MultiGel-21 (TOPBIO, Taiwan) under UV light. The GRA7-nPCR products of all DNA samples were sent for sequencing.

**Table 4:** Primer pairs and expected PCR product size for GRA7 nPCR and B1 nPCR

Target Gene	PCR	Primer paris (5′-3′)	Expected Size of PCR product
GRA7^a^	1st PCR	GRA7-FE1: CAAGCACCCGTTGACAGTCTCAG	316 bp
		GRA7-RE1: ACGATGCACCCATACCAACAGCCG	
	2nd PCR	GRA7-FI1: CACCACCAGCATGGATAAGGCATC	222 bp
		GRA7-RI1: CGGCGA GCTTCTTCAGCAAGTCT	
B1^b^	1st PCR	Outter-F: CTTTGAATCCCAAGCAAAACATGAG	400 bp
		Outter-R: GCGAGCCAAGACATCCATTGCTGA	
	2nd PCR	Inner-F: GTGATAGTATCGAAAGGTAT	220 bp
		Inner-R: ACTCTCTCTCAAATGTTCCT	

^a^ Primers were modified from previous study [18]

^b^ Primers were based on previous study [26]

## Supplementary Information

**Additional file 1.** 

## Data Availability

The datasets generated and/or analyzed during the current study are not publicly available due to internal regulations but are available from the corresponding author on reasonable request.

## Abbreviations

ELISA: Enzyme-linked immunosorbent assay
LAT: Latex agglutination test
nPCR: Nested polymerase chain reaction
GRA7: Dense granule protein 7
LC: *Lemur catta*
VV: *Varecia variegate*
Cy: *Cynomys*
PTT: *Panthera tigris tigris*
P: Positive
N: Negative
D: Doubtful
AP: Adjusted positive after LAT assay
AN: Adjusted negative after LAT assay
