# Supplementary figures and images for: Investigation of Toxoplasma infection in zoo animals using multispecies ELISA and GRA7 nested PCR
Source: BMC Vet Res. 2022 Sep 6;18:335. doi: 10.1186/s12917-022-03425-y (PMC9447357; doi:10.1186/s12917-022-03425-y)

1<sup>st</sup> PCR

2<sup>nd</sup> PCR

GRA7

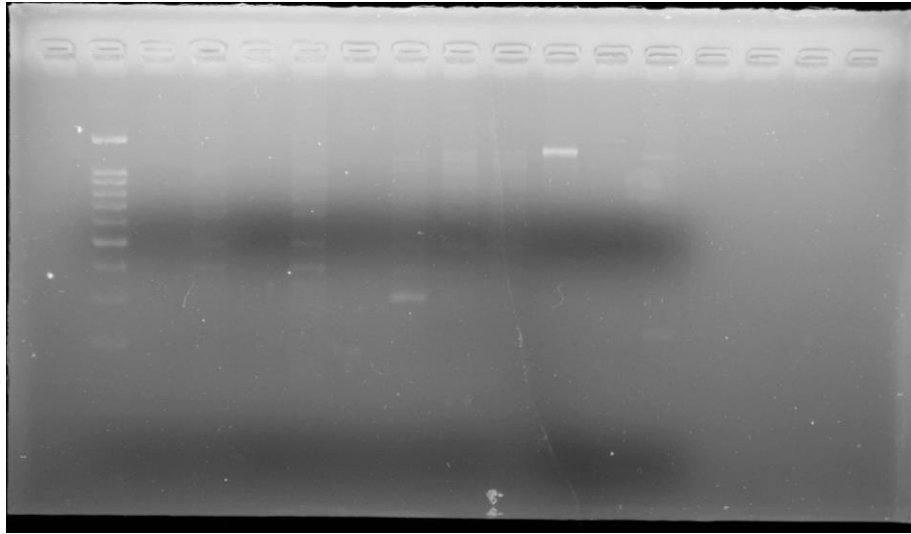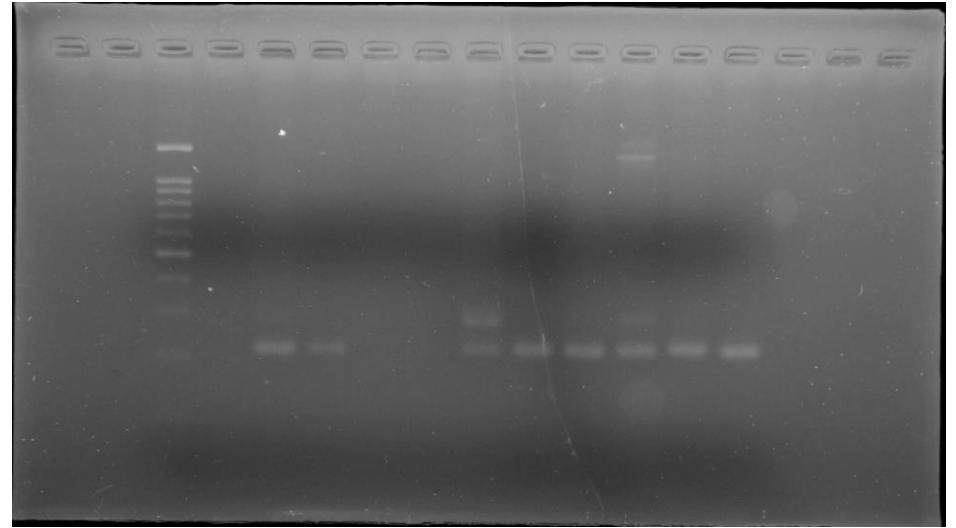

B1

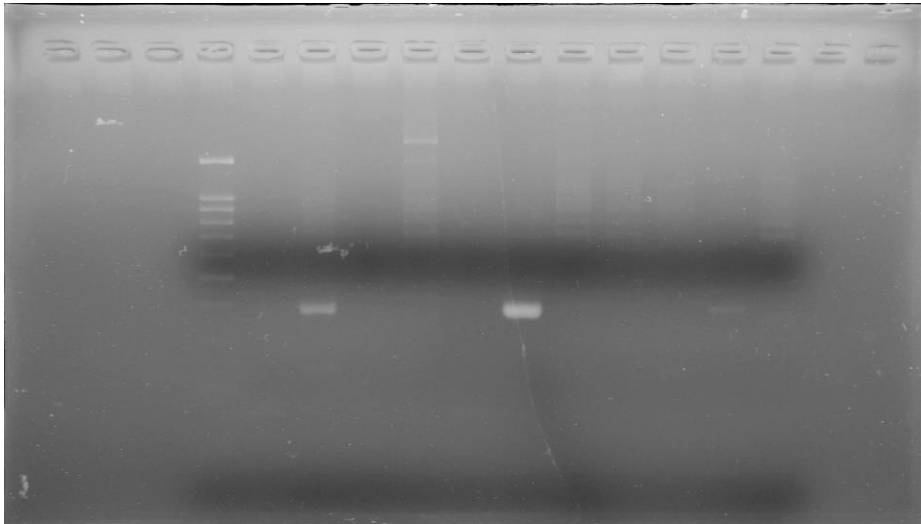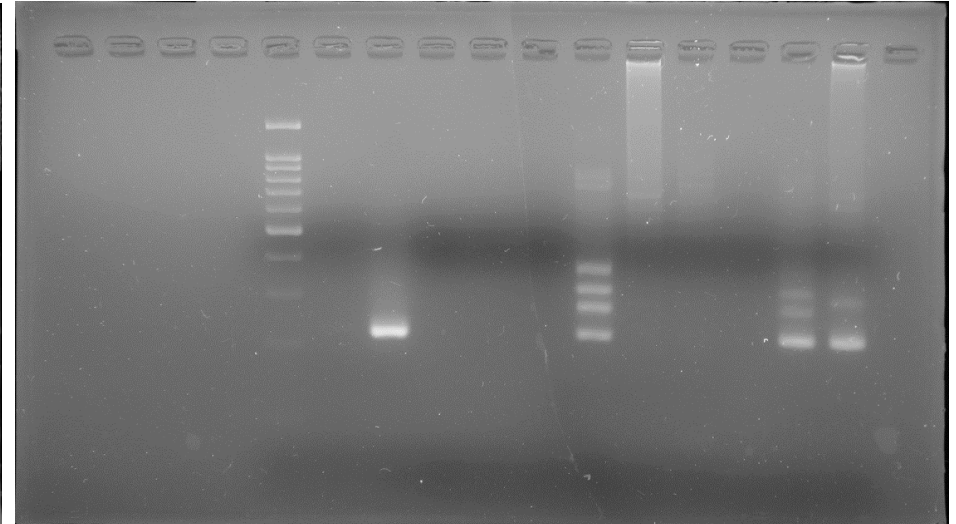

Supplement: Supplementary file 1 — Additional file 1. [file 12917_2022_3425_MOESM1_ESM.pdf]
